# Effect of 6-Shogaol Derived from Ginger (*Zingiber officinale*) on Dual-Species Biofilm Formation by *Streptococcus mutans* and *Candida albicans*

**DOI:** 10.3390/nu17182999

**Published:** 2025-09-19

**Authors:** Eun-Ha Jung, Geelsu Hwang, Ki-Rim Kim

**Affiliations:** 1 Departments of Dental Hygiene, Kyungpook National University, 2559 Gyeongsang-daero, Sangju 37224, Republic of Korea; jeunha725@knu.ac.kr; 2 Department of Preventive and Restorative Sciences, School of Dental Medicine, University of Pennsylvania, Philadelphia, PA 19104, USA; geelsuh@upenn.edu

**Keywords:** 6-shogaol, biofilm, *Candida albicans*, *Streptococcus mutans*

## Abstract

Background/Objectives: Dental plaque, a biofilm composed of accumulated oral microorganisms, is a key contributor to various oral diseases. 6-shogaol, a bioactive compound of ginger, is known to have pharmacological activities, including anticancer, anti-inflammatory, and antimicrobial activities. Therefore, we aimed to determine the effects of 6-shogaol on dual-species biofilms of *Streptococcus mutans* (*S. mutans*) and *Candida albicans* (*C. albicans*). Methods: Dual-species oral biofilms were formed on hydroxyapatite (HA) disks for 42 h and exposed to 6-shogaol. The pH was measured in the experimental medium, and the biomass, colony-forming unit (CFU) of microbial cells, and insoluble extracellular polysaccharides (EPS) were quantified in the biofilm formed on the HA disk. Confocal laser scanning microscopy (CLSM) was used to assess biofilm morphology, and quantitative polymerase chain reaction was performed to analyze *gtf* gene expression. Results: 6-shogaol dose-dependently reduced insoluble EPS, CFU counts, and dry weight of biofilms. The pH was maintained above 5.5 in the 6-shogaol-treated group. CLSM images showed that *S. mutans* proliferation, *C. albicans* hyphal development, and EPS production were markedly inhibited in biofilms treated with 6-shogaol. The expression of *gtfB* and *gtfC* was significantly downregulated by 6-shogaol. Conclusions: These findings suggest that 6-shogaol has the potential to be a promising natural product for the prevention and management of oral biofilm-related oral diseases.

## 1. Introduction

Dental plaque is a major factor in causing oral diseases such as caries and periodontal disease, significantly affecting oral health. This dental plaque is a complex microbial community composed of diverse oral microorganisms and exhibits potent pathogenicity through its interactions and metabolic activities [1]. The human oral cavity harbors over 700 species of microorganisms and some of these contribute to the formation and maturation of biofilms [2]. In particular, *Streptococcus mutans* (*S. mutans*) is the primary causative agent of cariogenic biofilm formation, synthesizing large amounts of extracellular polysaccharides (EPS), such as glucan and fructan, from sucrose using glucosyltransferase (Gtf) and fructosyltransferase (Ftf) enzymes. EPS is a key component of biofilm formation, contributing to the development and enhanced pathogenicity of cariogenic biofilms through strong surface adhesion, as well as acid production and tolerance [3,4].

*Candida albicans* (*C. albicans*), a fungus commonly found in the oral cavity, is known to cause oral candidiasis and other opportunistic infections [5]. As a major opportunistic pathogen, *C. albicans* increases the risk of various oral diseases, including caries, and persistent infections in immunosuppressed hosts and in poor oral environments [6]. Its pathogenicity is also closely related to various virulence mechanisms, including biofilm formation, tissue invasion, immune evasion, and the development of antifungal resistance [7]. Although *C. albicans* has limited ability to adhere to tooth surfaces or form its own biofilm, previous studies have reported that interactions with other microorganisms enhance the pathogenicity of biofilms. A study by Hwang et al. confirmed that the cell wall mannans of *C. albicans* bind with *gtfB* secreted by *S. mutans*, enabling glucan-mediated EPS structure formation on the fungal cell surface [8]. This EPS matrix not only contributes to the structural stability of the biofilm but also enhances resistance to antifungal agents [9]. Furthermore, Klein et al. demonstrated that the presence of sucrose and starch increase the expression of the *gtfB* gene in *S. mutans*, thereby increasing biofilm integrity [10]. Due to this synergistic interaction, the dual-species biofilm of *S. mutans* and *C. albicans* exhibit higher pathogenicity and antimicrobial resistance; moreover, it produces acid more rapidly and creates a denser EPS structure than its single-species counterparts [11].

Thus, controlling dual-species biofilms with conventional antibacterial agents (e.g., 0.2% chlorhexidine) or antifungal agents (e.g., fluconazole) can pose challenges [12,13]. Moreover, the widespread use of conventional chemical antibiotics has led to the emergence of drug-resistant strains and oral microbiota dysbiosis. Accordingly, biofilm control methods utilizing bioactive compounds from natural products with low toxicity and high biocompatibility are attracting attention [14].

6-shogaol, a bioactive compound produced during the drying process of *Zingiber officinale* (ginger), has been reported to exhibit various pharmacological properties, including antioxidant, anticancer, anti-inflammatory, and antimicrobial activities [15,16]. Ginger extract exerts significant antibacterial effect against *S. mutans* by inhibiting glucan synthesis and biofilm formation [17]. In particular, among the ginger components, 6-shogaol significantly suppressed *C. albicans* biofilm formation and hyphal transition [16].

Therefore, this study aims to elucidate the effect of 6-shogaol on the formation of *S. mutans–C. albicans* dual-species biofilm and evaluate its potential as a natural product-based therapeutic candidate for biofilm control.

## 2. Materials and Methods

### 2.1. Chemicals, and Microbial Species and Culture

**Figure 1:**
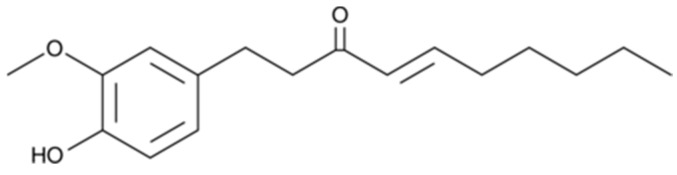
The chemical structure of 6-shogaol.

6-Shogaol (≥98% purity, Figure 1) was purchased from Cayman Chemical (Ann Arbor, MI, USA) and dissolved in dimethyl sulfoxide (Sigma-Aldrich, St. Louis, MO, USA).

*Streptococcus mutans* UA159 and *Candida albicans* SC5314 were used for biofilm experiments as described previously [18]. *S. mutans* and *C. albicans* were cultured aerobically in Brain Heart Infusion (BHI) and Yeast Mold (YM) culture medium (BD Difco, Franklin Lakes, NJ, USA) containing 1% glucose, respectively. After overnight incubation, the microorganisms were diluted 10-fold in each fresh culture medium and grown to mid-exponential phase. The optical density (OD) at 600 nm, in the range of 0.4–0.6, was measured using a microplate reader (Thermo Fisher Scientific, Waltham, MA, USA) for subsequent experiments.

### 2.2. Saliva Sample Preparation

Saliva samples were collected from healthy subjects who had taken no medication for at least 1 week, had fasted for 8 h, and had not used any oral hygiene products (such as mouthwash). This protocol was approved by the Institutional Review Board of Kyungpook National University (No. 2023-0246), and all subjects provided written informed consent. Whole saliva samples obtained by chewing paraffin were collected in sterile tubes on ice, centrifuged at 5500× *g* for 10 min at 4 °C, and the supernatant was filtered through a 0.22 μm polyethersulfone membrane filter (Millipore, Billerica, MA, USA). Filtered saliva samples were stored at 4 °C until experimentation.

### 2.3. Biofilm Formation Assay

The biofilm formation method was based on previously described models [18]. Hydroxyapatite (HA) disks (1.25 cm in diameter, surface area of 2.7 ± 0.2 cm^2^) were produced directly and placed vertically into the wells of a 24-well plate filled with saliva samples. The plate was mounted on an orbital shaker and shaken at 37 °C for 2 h to coat the saliva onto the HA disks. To prepare media for biofilm formation, filtered saliva samples containing 1% sucrose were added to 24-well plates and inoculated with *S. mutans* (1 × 10^6^ CFU/mL) and *C. albicans* (1 × 10^4^ CFU/mL). The experimental groups were treated with various concentrations of 6-shogaol. HA disks coated with saliva-derived pellicle were transferred to the prepared 24-well plate and cultured in an incubator with 5% CO_2_ at 37 °C. Fresh saliva medium containing 1% sucrose was replaced every 14 h. At the second media change, 28 h later, the pH of the conditioned medium was measured using an Orion Star™ A211 pH Benchtop Meter (Thermo Fisher Scientific). After a total of 42 h of incubation, the morphology of biofilm formed on HA disks was examined using confocal laser scanning microscopy (CLSM). For quantitative analysis, biofilms were removed from HA disks and homogenized by sonication in sterile saline solution. The homogenized biofilm suspension was aliquoted for colony-forming unit (CFU) assay of microbial cells, dry weight measurement, insoluble EPS analysis, and quantitative polymerase chain reaction (qPCR).

### 2.4. Biochemical Biofilm Analysis

The aliquot of each homogenized suspension was centrifuged at 5500× *g* for 10 min at 4 °C. The pellet was washed with sterile distilled water and dried in an oven. The weight of the dried biofilm was measured. Quantification of EPS was performed using an established colorimetric assay described previously [19]. For extracting insoluble EPS, the pellet was added with 1 N NaOH (0.3 mL per 1 mg dry weight) and incubated for 2 h at 37 °C with shaking. The supernatant was collected by centrifugation (14,000 rpm, 10 min, 4 °C), and the pellet was resuspended in 1N NaOH. The process was repeated three times continuously to collect the supernatant. The total insoluble EPS content was determined by the phenol–sulfuric acid method.

### 2.5. Colony-Forming Unit (CFU) Assay

To determine the number of *S. mutans* and *C. albicans* per biofilm, an aliquot of each biofilm suspension was serially diluted 10-fold and spread onto Trypticase™ Soy Agar (TSA II) with 5% sheep blood plates (Thermo Fisher Scientific). After incubating the agar plates for 48 h at 37 °C, the colonies were individually counted based on the distinct morphological characteristics of each species: *S. mutans* and *C. albicans.*

### 2.6. Confocal Laser Scanning Microscopy (CLSM)

Biofilm-formed HA disks were gently washed with sterile saline and stained with various dyes (Thermo Fisher Scientific), and each confocal image was analyzed, as described previously [18]; SYTO 9 green-fluorescent nucleic acid stain (485/498 nm; Molecular Probes Inc., Eugene, OR, USA) for *S. mutans*, concanavalin A (ConA) conjugated with tetramethylrhodamine (555/580 nm; Molecular Probes, Inc., Eugene, OR, USA) for *C. albicans*, Alexa Fluor 647-dextran conjugate (647/668 nm; Molecular Probes Inc.) for EPS. To label fluorescent markers, HA disks were incubated in the dark for 15 min at room temperature, and biofilm images were obtained using a Zeiss LSM 800 confocal microscope (Carl Zeiss, Jena, Germany). Biofilm images of each HA disk were scanned from at least three areas, and the biovolume (μm^3^/μm^2^) of each three-dimensionally reconstructed image was quantified using COMSTAT2 (version 2.1) plugin in ImageJ [20].

### 2.7. qPCR Analysis

Total RNA from biofilms was extracted using a TRIzol reagent (Thermo Fisher Scientific). First-strand cDNA from 1 µg total RNA was synthesized using a PrimeScript 1st strand cDNA Synthesis Kit (Takara Bio Inc., Shiga, Japan), according to the manufacturer’s protocol. qPCR was performed using Power SYBR Green PCR Master Mix (Thermo Fisher Scientific) on a StepOnePlus^TM^ Real-Time PCR system (Thermo Fisher Scientific). The sequences of primers used are as follows: 16s rRNA (5′- ATC ACT AGT AGA TGG ACC TG -3′ forward, 5′- TGT ATC GTC GCC TTG GTA AG-3′ reverse); *gtfB* (5′- AGC AAT GCA GCC AAT CTA CAA AT-3′ forward, 5′- AGC AAC TTT GCC GTT ATT GTC A-3′ reverse); *gtfC* (5′- GGT TTA ACG TCA AAA TTA GCT GTA TTA GC-3′ forward, 5′- CTC AAC CAA CCG CCA CTG TT-3′ reverse). Relative gene expression was calculated by normalizing each *gtf* gene to the 16S rRNA gene.

### 2.8. Statistical Analysis

All experiments were repeated at least three times independently. Statistical analysis was performed using SPSS Statistics v.23.0 (IBM Inc., Chicago, IL, USA) with Student’s *t*-test or one-way analysis of variance (ANOVA), followed by Tukey’s post hoc test. The confidence interval was set at 95%.

## 3. Results

### 3.1. Effect of 6-Shogaol on Biochemical Properties and CFU Counts of Dual-Species Biofilm by S. mutans and C. albicans

**Figure 2:**
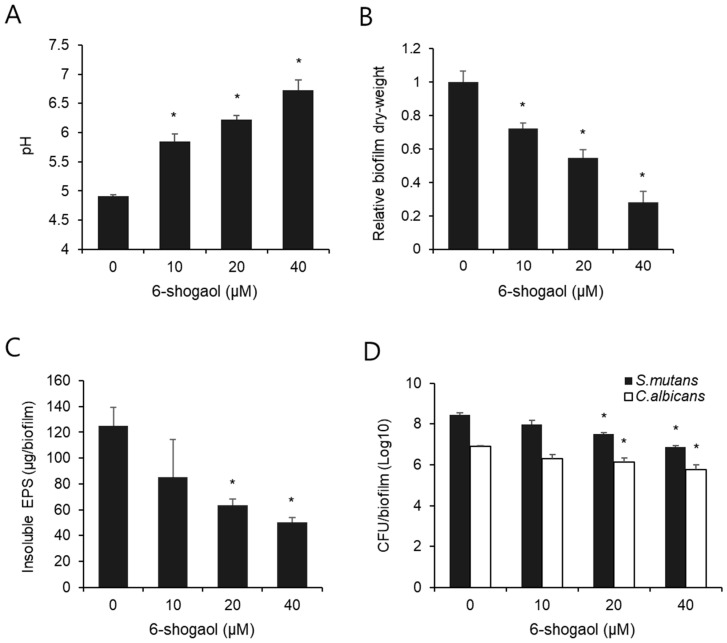
Biochemical properties and CFU counts of biofilm formed by *S. mutans* and *C. albicans* following treatment with 6-shogaol. (**A**) pH values of dual-species biofilm supernatants were measured at the middle (28 h) phase of biofilm formation. (**B**) The dry weight of each biofilm formed by *S. mutans* and *C. albicans* was measured and expressed as relative biofilm dry weight. (**C**) Amounts of insoluble EPS formed in the biofilm was quantified. (**D**) The number of viable cells of *S. mutans* and *C. albicans* in the biofilm was counted as CFU per biofilm. All data are presented as the mean ± standard deviation (SD) from at least three independent experiments. * *p* < 0.05 versus 0 μM 6-shogaol group.

As tooth demineralization occurs below pH 5.5, we measured the pH of the conditioned culture medium while replacing the second medium during the biofilm formation experiment. As shown in Figure 2A, the pH of the control biofilm supernatant with 0 μM 6-shogaol was approximately 4.9, which was lower than the critical pH of 5.5. However, the pH of the groups treated with 10 μM, 20 μM, and 40 μM 6-shogaol was 5.8, 6.2, and 6.7, respectively, all of which were higher than the critical pH 5.5.

After 42 h of the experiment, the biofilms formed by *S. mutans* and *C. albicans* were collected, and their dry weight was measured as the total biomass of each biofilm. As shown in Figure 2B, biofilm weight decreased in a dose-dependent manner in the group treated with 6-shogaol. Compared to the 0 μM 6-shogaol control group, the dry weight of biofilms was statistically significantly inhibited by approximately 27.9%, 45.3%, and 72.1% at concentrations of 10 μM, 20 μM, and 40 μM 6-shogaol, respectively.

We also measured the content of insoluble EPS produced within the biofilm and confirmed that it markedly decreased as the 6-shogaol concentration increased (Figure 2C). The insoluble EPS was statistically significantly reduced by more than about 50% at 6-shogaol concentrations of 20 μM or higher compared to the control group.

To determine the number of viable cells in the biofilm, the CFU of *S. mutans* and *C. albicans* were measured, respectively, and the results are shown in Figure 2D. Compared to the control group, the CFU of both *S. mutans* and *C. albicans* dose-dependently decreased in the biofilm group treated with 6-shogaol. In particular, 20 μM and 40 μM 6-shogaol significantly inhibited the CFU of both *S. mutans* and *C. albicans*.

### 3.2. Effect of 6-Shogaol on the Morphology of Dual-Species Biofilm by S. mutans and C. albicans

**Figure 3:**
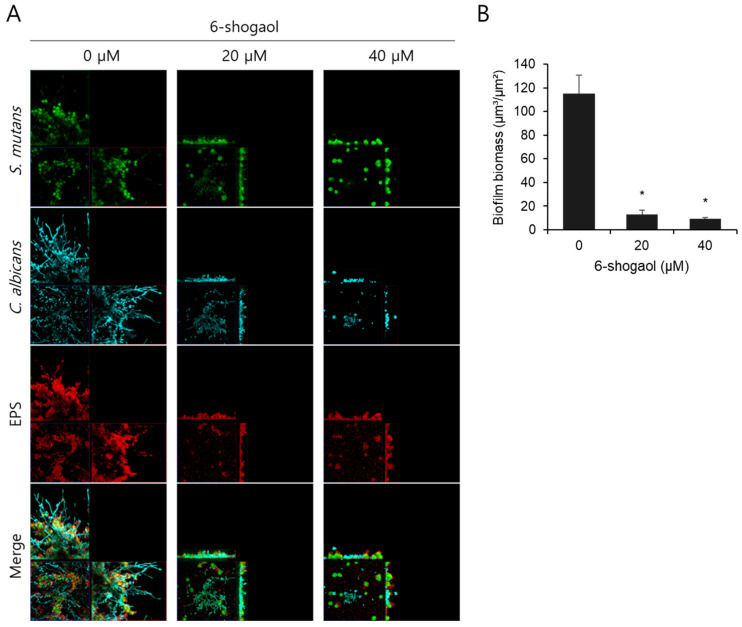
CLSM images of biofilm formed by *S. mutans* and *C. albicans* following treatment with 6-shogaol. (**A**) Biofilms were stained with fluorescent dyes to visualize different components: SYTO 9 (green) for *S. mutans*, concanavalin A-tetramethylrhodamine (Cyan) for *C. albicans*, and Alexa Fluor 647 (red) for EPS. The merged images show the spatial distribution and interaction of cells and EPS. Each panel presents XY (bottom left), XZ (top left), and YZ (bottom right) orthogonal views of the biofilm structure. (**B**) Total biofilm biomass was quantified and dataare presented as the mean ± SD from at least three independent experiments. * *p* < 0.05 versus 0 μM 6-shogaol group.

We observed the morphology and pathogenic characteristics of biofilms following treatment with 20 μM and 40 μM 6-shogaol and found statistically significant effects based on the results of microbiological and biochemical analyses of the biofilms. The morphology of biofilms formed by *S. mutans* and *C. albicans* on HA disks was visualized using confocal laser scanning microscopy, and tertiary structure images were obtained (Figure 3). The biofilm of the 0 μM 6-shogaol group exhibited amplified proliferation of *S. mutans* (green) and mycelial formation in *C. albicans* (cyan), forming microcolonies. EPS (red) was also excessively produced, resulting in the formation of a markedly thick and compact biofilm matrix. However, the group treated with 6-shogaol showed significantly reduced growth of *S. mutans* and hyphae formation in *C. albicans*; EPS production was also remarkably inhibited. The suppression of EPS synthesis led to a considerable decrease in overall biofilm thickness, as evident in the tertiary structural images. The total biomass of biofilms, quantified by COMSTAT analysis, was significantly reduced by 6-shogaol treatment. This inhibitory effect was more pronounced in the group treated with 40 μM compared to 20 μM 6-shogaol.

### 3.3. Effect of 6-Shogaol on the Expression of gtf Genes in Dual-Species Biofilm by S. mutans and C. albicans

**Figure 4:**
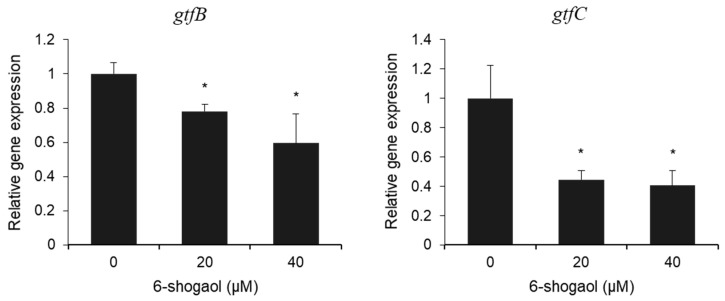
Relative gene expression levels of *gtfB* and gtfC in biofilm formed by *S. mutans* and *C. albicans* following treatment with 6-shogaol. Gene expression levels were measured using qPCR and normalized to 16S rRNA levels. Data are presented as the mean ± SD from at least three independent experiments. * *p* < 0.05 versus 0 μM 6-shogaol group.

To investigate the changes in *gtfs*, a sugar-fermenting enzyme that plays a key role in EPS synthesis by *S. mutans*, the expression levels of *gtfB* and *gtfC* genes were determined using qPCR analysis. In the 6-shogaol-treated groups, the relative gene expression of *gtfB* and *gtfC* statistically decreased in a concentration-dependent manner compared to the control group (Figure 4). Thus, the results revealed that 6-shogaol has an inhibitory effect on the expression of *gtf* genes in *S. mutans*, despite the presence of *C. albicans*.

## 4. Discussion

This study aimed to investigate the inhibitory effects of 6-shogaol on *S. mutans–C. albicans* dual-species biofilms and propose its potential as a natural product-based therapeutic agent for controlling oral biofilms. Our results showed that 6-shogaol significantly reduced the production of EPS, dry weight, and CFU in a concentration-dependent manner. We also observed that 6-shogaol mitigated the drop in pH below the critical threshold of 5.5, at which tooth demineralization begins. These findings indicate that 6-shogaol can inhibit oral biofilm formation and pathogenicity from multiple perspectives.

One of the key effects of 6-shogaol was a significant decrease in EPS production (Figure 2C). EPS forms the structural framework of biofilms, acting as a primary protective barrier that shields bacteria and fungi from the external environment, increasing their resistance to antibacterial and antifungal agents [21]. In this study, EPS production decreased with increased concentrations of 6-shogaol, with a significant difference observed at concentrations of 20 μM and above. This finding suggests that 6-shogaol can weaken the mechanical stability and reduce the pathogenicity of biofilms [22]. This result was confirmed by the morphology and pathogenic characteristics observed using CLSM (Figure 2). In the untreated control group, the interaction between the two species promoted bacterial growth, hyphal development, and excessive EPS production, with the formation of a thick and dense biofilm structure. In contrast, the 6-shogaol-treated groups showed significantly suppressed growth of *S. mutans* and hyphal formation in *C. albicans*, and EPS production also showed a notable decrease. This finding is illustrated by the differences in biomass between groups, where the inhibition of cell growth and EPS synthesis led to a reduction in biomass in the 6-shogaol-treated groups (Figure 2). The coexistence of *S. mutans* and *C. albicans* enhances biofilm growth and stability, forming more EPS and cellular mass than single-species biofilms [11,23]. The observed reduction in biomass suggests that there is a disruption in interspecies synergism. In the untreated group, insoluble glucans produced by *S. mutans* probably combined with cell wall components of *C. albicans* to form a structural scaffold, granting the dual-species biofilm high mechanical stability and resistance. Conversely, in the 6-shogaol-treated groups, the interaction between the species was possibly inhibited due to suppressed EPS production [8,24].

Furthermore, our qPCR analysis confirmed that 6-shogaol inhibited the expression of *gtfB* and *gtfC* (Figure 4). Both *gtfB* and *gtfC* are major contributors to biofilm pathogenicity and structural EPS formation. In particular, gtfB plays a pivotal role in creating highly pathogenic biofilms with mechanical stability and acid resistance by producing insoluble glucans that provide strong adhesion to the EPS matrix and the tooth surface [25]. Our results, which show that 6-shogaol inhibited *gtfB* and *gtfC*, suggest that it disrupts EPS matrix formation by blocking insoluble glucan synthesis [8,26]. Therefore, 6-shogaol acts by degrading pre-formed EPS and fundamentally weakening the biofilm structure at its foundation by inhibiting EPS synthesis.

In addition to these findings, previous studies have reported that 6-shogaol exerts antimicrobial and antibiofilm effects through multiple mechanisms, including disruption of microbial cell membranes, inhibition of quorum-sensing pathways, and suppression of biofilm- and hypha-related gene expression in *C. albicans* [16]. Chemically, 6-shogaol contains an α, β-unsaturated carbonyl group (Michael acceptor) and a phenolic hydroxyl group. The Michael acceptor moiety is reactive toward nucleophiles such as cysteine thiols, providing a plausible basis for covalent modification of microbial proteins [27], while the phenolic group and lipophilic side chain facilitate interactions with microbial membranes.

The inhibition of EPS formation could contribute to the decrease in dry weight and CFU counts (Figure 2B,D). As mentioned earlier, the effective suppression of EPS production, a primary component of biofilms, can be interpreted as 6-shogaol inhibiting the overall structural maturation of the biofilm while simultaneously limiting the attachment and survival of bacteria and fungi within [28]. Furthermore, previous research has shown that *S. mutans* and *C. albicans* dual-species biofilms form a mutualistic relationship, leading to higher dry weight, CFU counts, and EPS production than single-species biofilms [29]. Thus, the reduction in CFU observed in the 6-shogaol-treated group reflects suppressed bacterial growth, disrupted inter-species interactions, and inhibited biofilm colony formation [30].

The suppression of bacterial growth by 6-shogaol was also evident from the mitigated decrease in pH (Figure 2C). Generally, *S. mutans* metabolizes carbohydrates to produce acid, creating a low-pH environment that promotes tooth demineralization [31,32]. In our study, the control group (0 μM 6-shogaol) showed a pH of approximately 4.9, whereas the 6-shogaol-treated groups showed a pH range of 5.8 to 6.7, indicating a significant suppression of the acidification process. Considering that tooth demineralization occurs below the critical pH, this finding implies that 6-shogaol can effectively inhibit acidic environments, the primary cause of dental caries.

Oral biofilms are composed of multiple species, and traditional antimicrobial and antifungal agents, such as chlorhexidine or fluconazole, have limitations in effectively controlling these biofilms, leading to the demand for new approaches using natural substances or non-nutritive sweeteners [33,34]. From this perspective, 6-shogaol, a natural compound derived from ginger, exhibits various significant effects on both cells and bacteria [15,16]. Although oral biofilms are polymicrobial in nature, the effects of 6-shogaol have been evaluated on single species. The results of this study demonstrate various antimicrobial effects on a multi-species biofilm, showing its potential as a new natural product-based candidate for preventing oral diseases.

While this study demonstrates the inhibitory effects of 6-shogaol on oral biofilms, it has some limitations regarding its clinical application, as the present work was conducted in vitro. Future research should include preclinical studies employing microcosm biofilm models that more actually reflect the complexity of the oral environment, followed by carefully designed clinical trials to further assess the biosafety as well as the therapeutic efficacy of 6-shogaol. Furthermore, to strengthen its potential as a plant-derived antimicrobial therapeutic, it would be valuable to investigate the activity of 6-shogaol against pre-formed biofilms. In this regard, subsequent research could explore the impact of 6-shogaol on pre-formed dual- or multi-species biofilms and extend these findings by validating its antimicrobial effects in appropriate in vivo models. Such investigations may not only provide more clinically meaningful and comprehensive insights into the therapeutic potential of 6-shogaol but also contribute to a broader understanding of how plant-derived compounds can be integrated into oral care strategies for the prevention and treatment of oral biofilm-related diseases.

## 5. Conclusions

This study confirmed that 6-shogaol inhibited the formation and pathogenicity of dual-species biofilms by *S. mutans* and *C. albicans*. Based on these findings, 6-shogaol can be used as a foundational resource for developing natural product-based biofilm control strategies for prevention and treatment of biofilm-related oral disease.

## Data Availability

The raw data supporting the conclusions of this article will be made available by the authors on request.

## Abbreviations

The following abbreviations are used in this manuscript:

*S. mutans*	*Streptococcus mutans*
EPS	Extracellular polysaccharides
*C. albicans*	*Candida albicans*
HA	Hydroxyapatite
CLSM	Confocal laser scanning microscopy
CFU	Colony-forming unit
qPCR	quantitative polymerase chain reaction
SD	Standard deviation

## References

[1] Bowen W.H., Burne R.A., Wu H., Koo H. (2018). Oral Biofilms: Pathogens, Matrix, and Polymicrobial Interactions in Microenvironments. Trends Microbiol..

[2] Valm A.M. (2019). The Structure of Dental Plaque Microbial Communities in the Transition from Health to Dental Caries and Periodontal Disease. J. Mol. Biol..

[3] Zhang Q., Ma Q., Wang Y., Wu H., Zou J. (2021). Molecular mechanisms of inhibiting glucosyltransferases for biofilm formation in Streptococcus mutans. Int. J. Oral Sci..

[4] Paes Leme A.F., Koo H., Bellato C.M., Bedi G., Cury J.A. (2006). The role of sucrose in cariogenic dental biofilm formation--new insight. J. Dent. Res..

[5] Vila T., Sultan A.S., Montelongo-Jauregui D., Jabra-Rizk M.A. (2020). Oral Candidiasis: A Disease of Opportunity. J. Fungi.

[6] Lu S.Y. (2021). Oral Candidosis: Pathophysiology and Best Practice for Diagnosis, Classification, and Successful Management. J. Fungi.

[7] Macias-Paz I.U., Pérez-Hernández S., Tavera-Tapia A., Luna-Arias J.P., Guerra-Cárdenas J.E., Reyna-Beltrán E. (2023). Candida albicans the main opportunistic pathogenic fungus in humans. Rev. Argent. Microbiol..

[8] Hwang G., Liu Y., Kim D., Li Y., Krysan D.J., Koo H. (2017). Candida albicans mannans mediate Streptococcus mutans exoenzyme GtfB binding to modulate cross-kingdom biofilm development in vivo. PLoS Pathog..

[9] Kim D., Liu Y., Benhamou R.I., Sanchez H., Simón-Soro Á., Li Y., Hwang G., Fridman M., Andes D.R., Koo H. (2018). Bacterial-derived exopolysaccharides enhance antifungal drug tolerance in a cross-kingdom oral biofilm. ISME J..

[10] Klein M.I., Duarte S., Xiao J., Mitra S., Foster T.H., Koo H. (2009). Structural and molecular basis of the role of starch and sucrose in Streptococcus mutans biofilm development. Appl. Environ. Microbiol..

[11] Falsetta M.L., Klein M.I., Colonne P.M., Scott-Anne K., Gregoire S., Pai C.H., Gonzalez-Begne M., Watson G., Krysan D.J., Bowen W.H. (2014). Symbiotic relationship between Streptococcus mutans and Candida albicans synergizes virulence of plaque biofilms in vivo. Infect. Immun..

[12] Li Y., Huang S., Du J., Wu M., Huang X. (2023). Current and prospective therapeutic strategies: Tackling Candida albicans and Streptococcus mutans cross-kingdom biofilm. Front. Cell. Infect. Microbiol..

[13] Fan F., Liu Y., Liu Y., Lv R., Sun W., Ding W., Cai Y., Li W., Liu X., Qu W. (2022). Candida albicans biofilms: Antifungal resistance, immune evasion, and emerging therapeutic strategies. Int. J. Antimicrob. Agents.

[14] Chi Y., Wang Y., Ji M., Li Y., Zhu H., Yan Y., Fu D., Zou L., Ren B. (2022). Natural products from traditional medicine as promising agents targeting at different stages of oral biofilm development. Front. Microbiol..

[15] Huang H., Kim M.O., Kim K.R. (2021). Anticancer effects of 6-shogaol via the AKT signaling pathway in oral squamous cell carcinoma. J. Appl. Oral Sci..

[16] Lee J.H., Kim Y.G., Choi P., Ham J., Park J.G., Lee J. (2018). Antibiofilm and Antivirulence Activities of 6-Gingerol and 6-Shogaol Against Candida albicans Due to Hyphal Inhibition. Front. Cell. Infect. Microbiol..

[17] Hasan S., Danishuddin M., Khan A.U. (2015). Inhibitory effect of zingiber officinale towards Streptococcus mutans virulence and caries development: In vitro and in vivo studies. BMC Microbiol..

[18] Kim H.E., Liu Y., Dhall A., Bawazir M., Koo H., Hwang G. (2020). Synergism of Streptococcus mutans and Candida albicans Reinforces Biofilm Maturation and Acidogenicity in Saliva: An In Vitro Study. Front. Cell. Infect. Microbiol..

[19] de Sousa D.L., Lima R.A., Zanin I.C., Klein M.I., Janal M.N., Duarte S. (2015). Effect of Twice-Daily Blue Light Treatment on Matrix-Rich Biofilm Development. PLoS ONE.

[20] Heydorn A., Nielsen A.T., Hentzer M., Sternberg C., Givskov M., Ersbøll B.K., Molin S. (2000). Quantification of biofilm structures by the novel computer program COMSTAT. Microbiology.

[21] Li H., Liu H., Zhang L., Hieawy A., Shen Y. (2023). Evaluation of extracellular polymeric substances matrix volume, surface roughness and bacterial adhesion property of oral biofilm. J. Dent. Sci..

[22] Cugini C., Shanmugam M., Landge N., Ramasubbu N. (2019). The Role of Exopolysaccharides in Oral Biofilms. J. Dent. Res..

[23] Xiao J., Zeng Y., Rustchenko E., Huang X., Wu T.T., Falsetta M.L. (2023). Dual transcriptome of Streptococcus mutans and Candida albicans interplay in biofilms. J. Oral Microbiol..

[24] Ellepola K., Liu Y., Cao T., Koo H., Seneviratne C.J. (2017). Bacterial GtfB Augments Candida albicans Accumulation in Cross-Kingdom Biofilms. J. Dent. Res..

[25] Koo H., Xiao J., Klein M.I., Jeon J.G. (2010). Exopolysaccharides produced by Streptococcus mutans glucosyltransferases modulate the establishment of microcolonies within multispecies biofilms. J. Bacteriol..

[26] Yamashita Y., Bowen W.H., Burne R.A., Kuramitsu H.K. (1993). Role of the Streptococcus mutans gtf genes in caries induction in the specific-pathogen-free rat model. Infect. Immun..

[27] Bischoff-Kont I., Fürst R. (2021). Benefits of Ginger and Its Constituent 6-Shogaol in Inhibiting Inflammatory Processes. Pharmaceuticals.

[28] Koo H., Falsetta M.L., Klein M.I. (2013). The exopolysaccharide matrix: A virulence determinant of cariogenic biofilm. J. Dent. Res..

[29] Lobo C.I.V., Rinaldi T.B., Christiano C.M.S., De Sales Leite L., Barbugli P.A., Klein M.I. (2019). Dual-species biofilms of Streptococcus mutans and Candida albicans exhibit more biomass and are mutually beneficial compared with single-species biofilms. J. Oral Microbiol..

[30] Lemos J.A., Palmer S.R., Zeng L., Wen Z.T., Kajfasz J.K., Freires I.A., Abranches J., Brady L.J. (2019). The Biology of Streptococcus mutans. Microbiol. Spectr..

[31] Zero D.T., van Houte J., Russo J. (1986). Enamel demineralization by acid produced from endogenous substrate in oral streptococci. Arch. Oral Biol..

[32] Van Loveren C., Fielmich A.M., Ten Brink B. (1987). Comparison of the effects of fluoride and the ionophore nigericin on acid production by Streptococcus mutans and the resultant in vitro enamel demineralization. J. Dent. Res..

[33] Jeong G.J., Khan F., Tabassum N., Kim Y.M. (2024). Alteration of oral microbial biofilms by sweeteners. Biofilm.

[34] Gao Z., Chen X., Wang C., Song J., Xu J., Liu X., Qian Y., Suo H. (2023). New strategies and mechanisms for targeting Streptococcus mutans biofilm formation to prevent dental caries: A review. Microbiol. Res..

